# Endurance exercise training does not limit coronary atherosclerosis in familial hypercholesterolemic swine

**DOI:** 10.14814/phy2.14008

**Published:** 2019-02-26

**Authors:** Darla L. Tharp, Isabelle Masseau, Jan Ivey, Maurice Harold Laughlin, Douglas K. Bowles

**Affiliations:** ^1^ Department of Biomedical Sciences University of Missouri Columbia Missouri; ^2^ Department of Clinical Sciences Université de Montreal St‐Hyacinthe Canada; ^3^ Dalton Cardiovascular Research Center University of Missouri Columbia Missouri

**Keywords:** Angiography, atherosclerosis, coronary artery, exercise training, intravascular ultrasound, swine, treadmill running

## Abstract

Human studies demonstrate that physical activity reduces both morbidity and mortality of coronary heart disease (CHD) including decreased progression and/or regression of CHD with life‐style modification which includes exercise. However, evidence supporting an intrinsic, direct effect of exercise in attenuating the development of CHD is equivocal. One limitation has been the lack of a large animal model with clinically evident CHD disease. Thus, we examined the role of endurance exercise in CHD development in a swine model of familial hypercholesterolemia (FH) that exhibits robust, complex atherosclerosis. FH swine were randomly assigned to either sedentary (Sed) or exercise trained (Ex) groups. At 10 months of age, Ex pigs began a 10 months, moderate‐intensity treadmill‐training intervention. At 14 months, all pigs were switched to a high‐fat, high‐cholesterol diet. CHD was assessed by intravascular ultrasound (IVUS) both prior to and after completion of 6 months on the HFC diet. Prior to HFC diet, Ex resulted in a greater coronary artery size in the proximal and mid sections of the LCX compared to SED, with no effect in the LAD. After 6 months on HFC diet, there was a 5–6 fold increase in absolute plaque volume in all segments of the LCX and LAD in both groups. At 20 months, there was no difference in vessel volume, lumen volume, absolute or relative plaque volume in either the LCX or LAD between Sed and Ex animals. These findings fail to support an independent, direct effect of exercise in limiting CHD progression in familial hypercholesterolemia.

## Introduction

Despite lipid lowering and emerging anti‐inflammatory agents, atherosclerosis remains the leading cause of death in both men and women in the United States (Silvestre‐Roig et al. 2014; Mozaffarian et al. 2015). An estimated 16.5 million Americans and 2.4 million Canadians > 20 years of age have coronary heart disease (CHD) (Public Health Agency of Canada, 2017; Benjamin et al. 2018). The estimated total cost of heart disease in the United States alone for 2013–14 was >$329 billion (Benjamin et al. 2018). Thus, interventions that reduce CHD would have substantial health and economic benefit.

Increased physical activity has been proposed as a major beneficial intervention in reducing chronic disease, including CHD (Booth et al. 2017). Physical inactivity has been deemed a primary risk factor for cardiovascular disease in general, CHD (Thompson et al. 2003) and the beneficial effects of exercise and increased physical activity on cardiovascular morbidity and mortality are well documented (Thompson et al. 2003). Meta‐analysis of prospective cohort studies in humans demonstrate that moderate to high levels of physical activity reduce both morbidity and mortality of CHD (Thompson et al. 2003; Sofi et al. 2008). In addition, secondary prevention trials in humans have demonstrated a reduction in mortality (Thompson et al. 2003), with some showing a decreased progression and/or regression of coronary atherosclerosis with life‐style modification including exercise (Hambrecht et al. 1993; Niebauer et al. 1997).

However, a comprehensive review combining human and animal literature reveals equivocal evidence to support a direct, independent exercise effect in attenuating the development of coronary atherosclerosis (Laughlin et al. 2012). Exercise has been found to reduce development and/or cause regression of atherosclerotic lesions in some animal models of disease including mice (Okabe et al. 2006, 2007) and rabbits (Yang et al. 2003), however in larger mammals, including primates, the evidence is more equivocal. Over 40 years ago, Link et al. (1972) reported reduced atheroma in exercise‐trained swine, however more recent studies in swine have produced mixed results (Turk and Laughlin 2004; Long et al. 2010; Sturek 2011). A major limitation has been the lack of large animal models with clinically evident CHD disease, that is, pronounced plaque volume or lumen loss, as defined by angiography and/or intravascular ultrasound (IVUS) that more closely resembles clinically relevant disease. Thus, we sought to determine the role of endurance exercise in coronary atherosclerosis development and compensatory remodeling in a swine model of familial hypercholesterolemia (FH) that exhibits robust, complex atherosclerosis using IVUS. IVUS allows identification of plaque in angiographically non‐stenotic lesions, quantification of atheroma burden, assessment of arterial remodeling and three‐dimensional arterial reconstruction for volumetric analysis of plaque.

## Material and Methods

### Experimental animals

**Figure 1:**
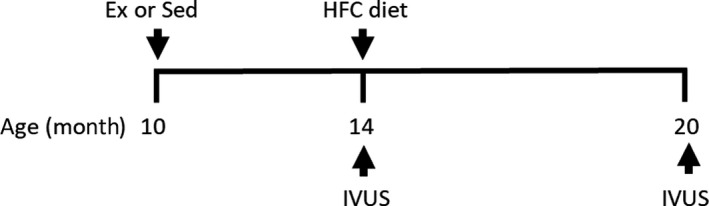
Overall study design. FH swine were randomly assigned to sedentary (Sed) or treadmill trained (Ex) groups at 10 months of age. At 14 months of age, both Sed and Ex groups underwent intravascular ultrasound (IVUS) and subsequently placed on a high‐fat, high‐cholesterol (HFC) diet. IVUS was also obtained after 6 months on diet (20 months of age).

Experimental protocols were approved by the University of Missouri Animal Care and Use Committee and in accordance with the “Principles for the Utilization and Care of Vertebrate Animals used in testing, Research and Training (Guide for the Care and Use of Laboratory Animals, 2011).” Castrated male Rapacz familial hypercholesterolemic (FH) swine were obtained from the University of Wisconsin Swine Research and Teaching Center. These swine are characterized by a single missense mutation in the LDL receptor that decreases its affinity for LDL, resulting in elevated total cholesterol levels (Hasler‐Rapacz et al. 1998). The overall study design is depicted in Figure 1.

### Endurance exercise training

Pigs were randomly assigned to either sedentary (Sed) or exercise trained (Ex) groups. Pigs in the sedentary group were restricted to normal cage activity. At 10 months of age, pigs in the exercise‐training group began a 10 months exercise‐training intervention consisting of moderate‐intensity (70% of maximal heart rate) aerobic exercise on treadmills, once per day, for 5 days each week as described previously (Bunker and Laughlin 2010; Company et al. 2010; Simmons et al. 2014). Briefly, the exercise protocol was short in duration and low intensity at the beginning of the intervention (5 min warm‐up at 2–2.5 m.p.h., 15 min at 4 m.p.h., 20 min at 3 m.p.h., and a 5 min cool‐down at 2–2.5 m.p.h.), but subsequently increased in difficulty such that, by week 10 of training, pigs exercised for 85 min each day, consisting of a 5 min warm‐up at 2–2.5 m.p.h., 15 min at 6.5–7 m.p.h., 60 min at 4.5–5 m.p.h., and a 5 min cool‐down at 2–2.5 m.p.h. This exercise protocol has been shown to result in typical endurance exercise training adaptations in FH swine, including increased heart weight to body weight ratio, and increased exercise tolerance during a graded exercise test (Bunker and Laughlin 2010).

### Diet intervention

Prior to 14 months of age, pigs were fed standard chow (by weight 16.7% protein, 2.6% fat, 53.2% carbohydrate; 2.57 kcal/g). At 14 months, all pigs were switched to a high‐fat, high‐cholesterol diet (HFC; by weight 13% protein, 21.3% fat, 41.4% carbohydrate, 2% cholesterol and 1% sodium cholate) as previously described (McCommis et al. 2011; de Beer et al. 2013; Bender et al. 2014, 2016). We have found that this protocol yields greater consistency in the development of coronary atherosclerosis than with the standard pig chow. Serum lipid analysis was performed on an Olympus AU400 chemistry analyzer by the Veterinary Medical Diagnostic Laboratory of the University of Missouri.

### Intravascular ultrasound (IVUS)

Coronary atherosclerosis was assessed by intravascular ultrasound (IVUS) both prior to (14 months of age) and after completion of 6 months on the HFC diet (20 months of age). Angiograms and IVUS were obtained using standard coronary catheterization techniques as described previously (Tharp et al. 2008, 2009; Fleenor and Bowles 2009a,2009b; Kilroy et al. 2014, 2015; Bender et al. 2016). Briefly, a 7F introducer was inserted in the right or left femoral artery. Heparin (300 U/kg) was administered following femoral access and maintenance doses given every hour (100 U/kg). A guide catheter (6F) was directed up the aorta and engaged into the left ostium under fluoroscopic guidance. The left main coronary artery was selectively engaged and IVUS pullbacks (0.5 mm/sec; Galaxy II, Boston Scientific, 40 MHz) were obtained for the proximal 3 cm of both the left anterior descending (LAD) and left circumflex (LCX) arteries after intracoronary nitroglycerin representing approximately one‐half to two‐thirds, respectively, of each vessel length. Three‐dimensional reconstruction of IVUS pullbacks was performed using QIvus software (Medis). The total segment was subdivided into three equal sections (10 mm each) and total vessel volume, lumen volume, total plaque volume, and percent plaque burden were determined in the proximal, mid, and distal segments as previous (Bender et al. 2016), that is, vessel volume = EEL area x length; lumen volume = lumen area x length; total plaque burden volume = (EEL area‐lumen area) x length; and percent plaque burden = total plaque volume/vessel volume.

### Statistical analysis

All data are presented as mean ± SE. Data from the 14 months (*n* = 7) and 20 months (*n* = 8) sedentary animals were from two separate studies, while in the exercise trained group both time points were obtained on the same animal (*n* = 8), thus the comparison between sedentary and exercise trained vessels was a cross‐sectional design. In two of the sedentary animals, IVUS data were collected on only one vessel due to technical issues. Two‐way ANOVA was used for between group comparisons at 14 and 20 months (segment x training) with significance defined as p < 0.05.

## Results

### Group characteristics

Body weights for sedentary and exercise‐trained groups were similar at both 14 months of age (65 ± 2 vs. 62 ± 2 kg, resp.) and 20 months of age (88 ± 3 vs. 87 ± 2 kg, resp.). Total cholesterol (TC), HDL, and LDL were significantly increased by the HFC diet while the total cholesterol to HDL ratio remained constant. At 20 months of age the TC levels were 4–5 fold greater than the designated optimum for humans (Grundy et al. 2018) and the TC/HDL ratio was approximately 2‐fold greater than the risk threshold level for both men and women (5.0 and 4.5, respectively)(Millan et al. 2009). While there was a trend for TC, HDL, and LDL levels to be somewhat elevated in the exercise trained group, consistent with prior studies in FH swine (McCommis et al. 2011; Simmons et al. 2014), exercise training had no significant effect on any lipid parameter (Table 1).

**Table 1:** Lipid characteristics

	14 months		20 months	
	Sed (*n* = 7)	Ex (*n* = 8)	Sed (*n* = 8)	Ex (*n* = 8)
Total cholesterol (TC; mg/dL)	460 ± 25	489 ± 37	802 ± 93*	1031 ± 56*
HDL (mg/dL)	49 ± 3	49 ± 3	77 ± 6*	90 ± 6*
LDL (mg/dL)	368 ± 20	396 ± 32	652 ± 80*	842 ± 43*
TC/HDL	9.6 ± 0.7	10.3 ± 1.0	10.6 ± 1.0	11.7 ± 0.9

* *P* < 0.05 versus 14 months.

### Coronary atherosclerotic lesion morphometry

**Figure 2:**
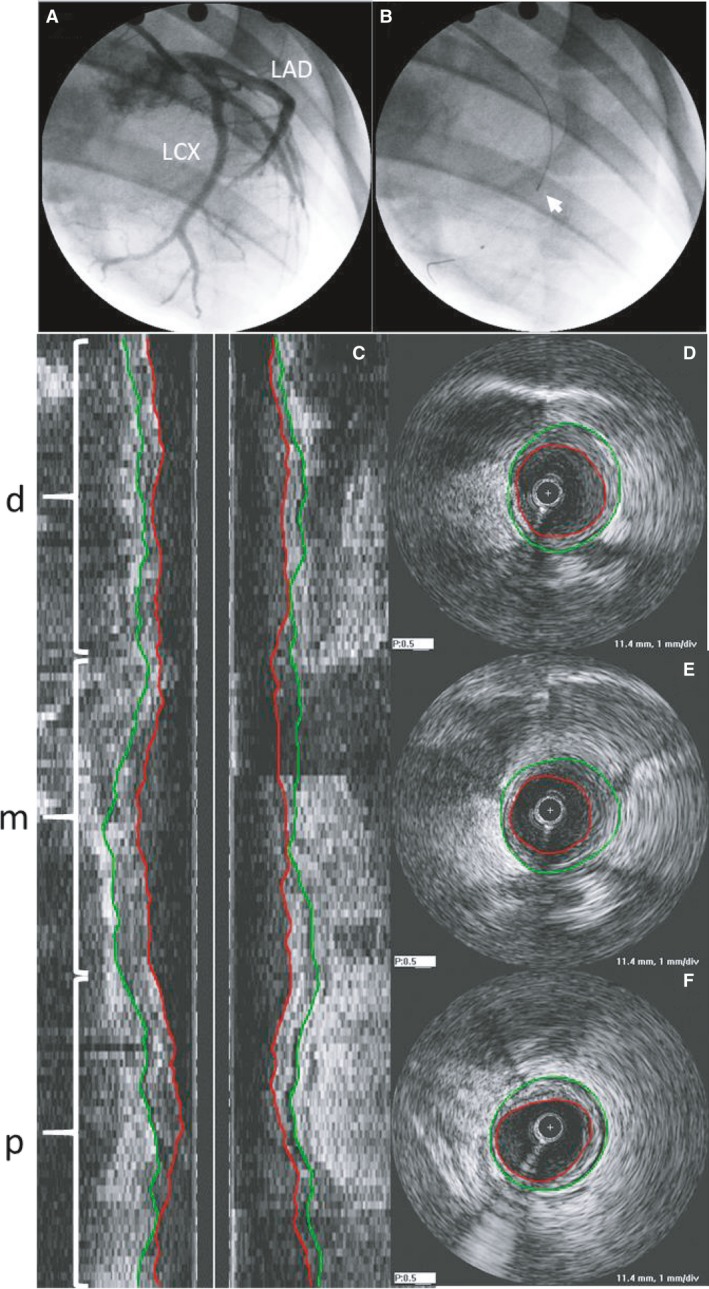
(A) Typical angiogram showing left circumflex (LCX) and left anterior descending (LAD) and (B) placement of IVUS prior to pullback (white arrow; RAO 60°) in a sedentary animal. (C) Longitudinal cross‐section of IVUS pullback in LCX indicating distal (*d*), mid (*m*) and proximal (*p*) sections with corresponding cross‐sectional images in distal (D), mid (E), and proximal (F). Green lines indicate vessel border (external elastic lamina; EEL) and red lines indicate luminal border.

**Figure 3:**
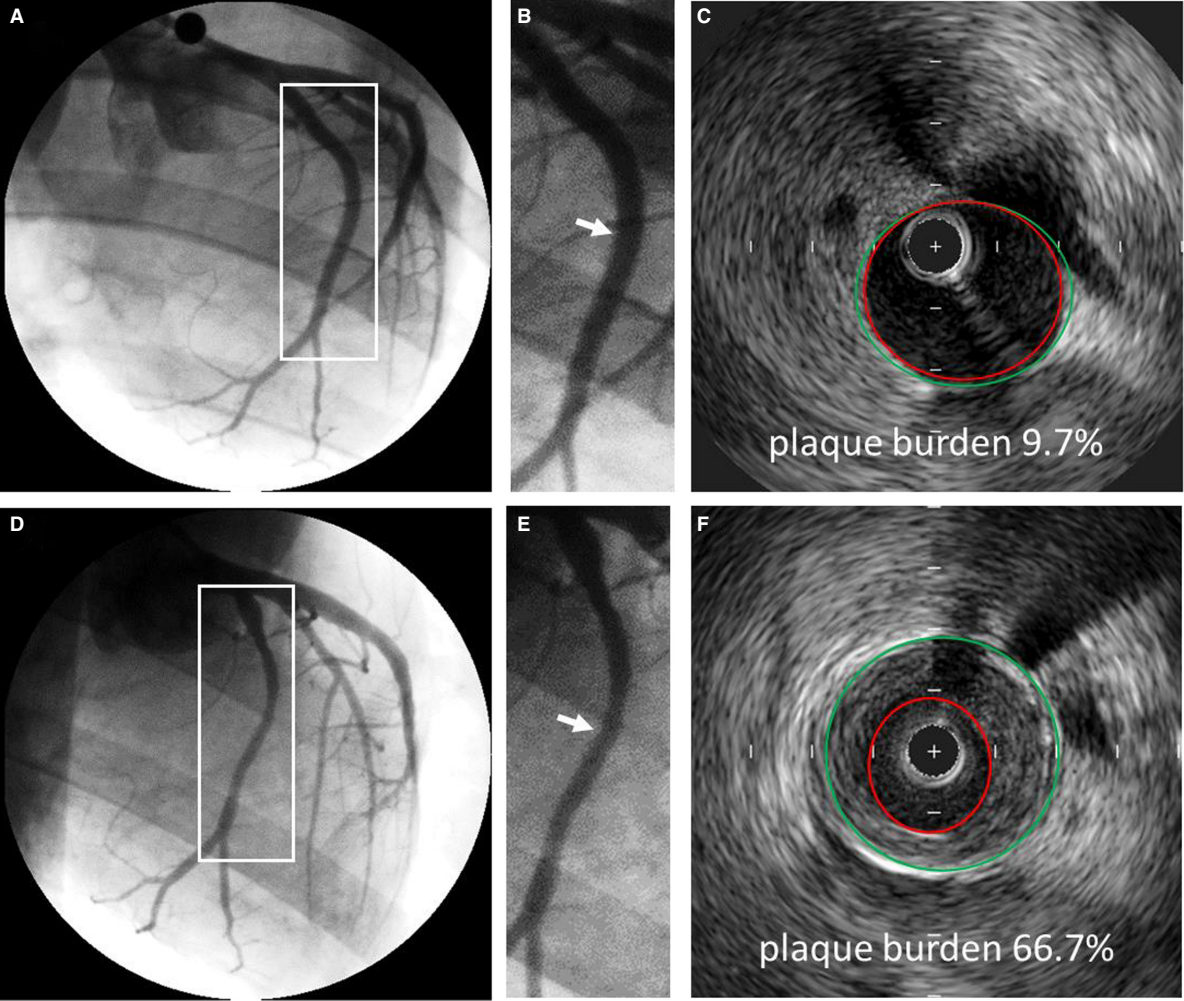
Representative angiograms and IVUS cross‐sectional images showing progression of CHD in the LCX of a sedentary animal. Angiograms of the LCX at 14 months (A) and 20 months (D). White boxes are magnified in B and E, respectively. White arrows indicate corresponding IVUS cross sections in C and F. Relative plaque (plaque burden) in the mid section of the LCX increased from 9.7% to 66.7%. Quantitative angiography determined that luminal diameter decreased from 2.99 (B) to 2.02 mm (E). Green lines indicate vessel border (external elastic lamina; EEL) and red lines indicate luminal border.

Six months of HFC diet resulted in significant coronary atherosclerosis in both the LCX and LAD. A representative angiogram and IVUS of the left circumflex artery from a sedentary animal is depicted in Figure 2. The high‐fat, high‐cholesterol diet in the FH pig produced a fairly uniform plaque throughout the proximal, mid, and distal segments. Figure 3 shows the development of coronary atherosclerosis in the LCX of a sedentary animal. At 14 months both the angiogram (A, B) and IVUS (C), demonstrated a lack of coronary disease. After 6 months on diet, there was visible non‐uniformity and focal narrowing of the LCX on angiogram (D, E) and development of significant relative plaque burden by IVUS (F; 66.7% in the mid LCX indicated by arrow in 3E).

**Figure 4:**
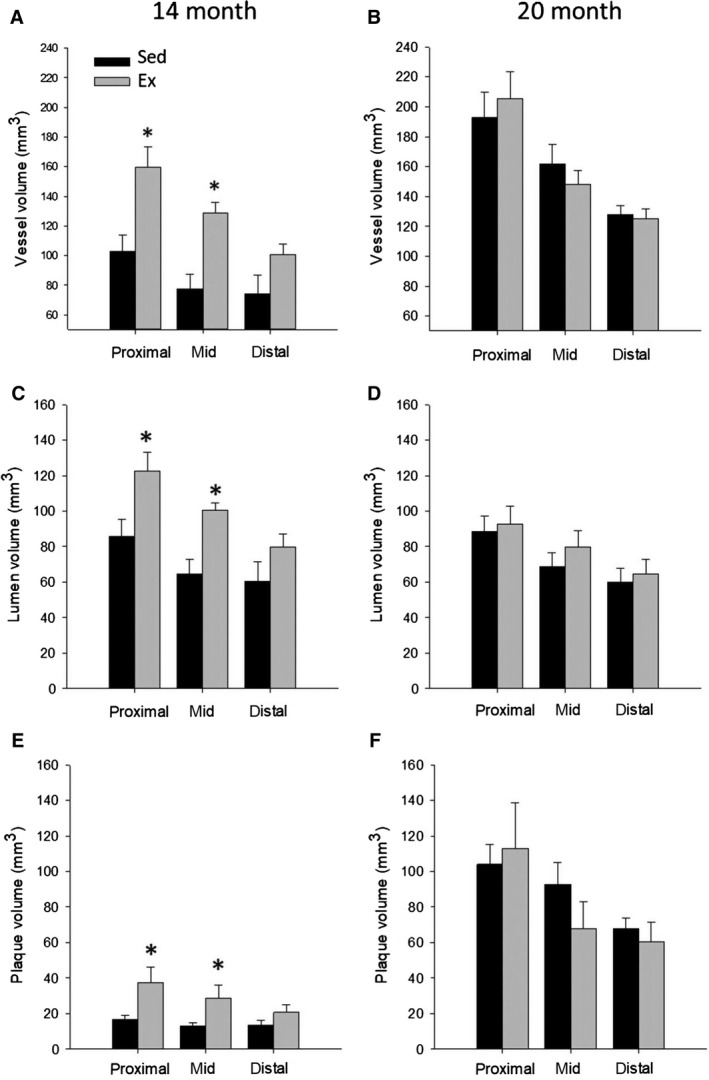
3D volumetric analysis of the proximal, mid, and distal sections of the left circumflex artery (LCX) in Sed and Ex at 14 (*n* = 6 and 8, resp. for all segments) and 20 months (*n* = 8 and 8, resp. for all segments). Prior to HFC, vessel, lumen and absolute plaque volume were significantly increased in Ex versus Sed for the proximal and mid sections. After 6 months on HFC diet, absolute plaque volume, and vessel volume increased, with no difference between Sed and Ex. *P < 0.05 vs. Sed.

**Figure 5:**
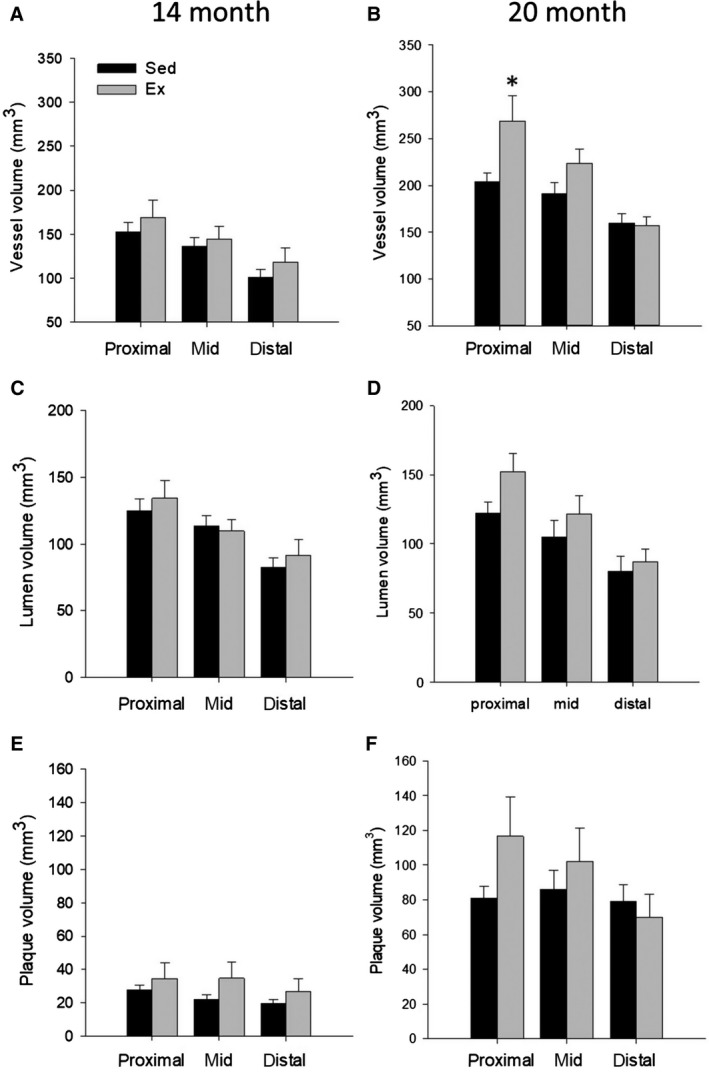
3D volumetric analysis of the proximal, mid, and distal sections of the left anterior descending artery (LAD) in Sed and Ex at 14 (*n* = 6 and 8, resp. for all segments) and 20 months (*n* = 8 and 8, resp. for all segments). There was no significant difference between Sed and Ex at 14 or 20 months for either absolute plaque, vessel, or lumen volume, except a greater vessel volume in the proximal LAD in Ex. *P < 0.05 vs. Sed.

**Figure 6:**
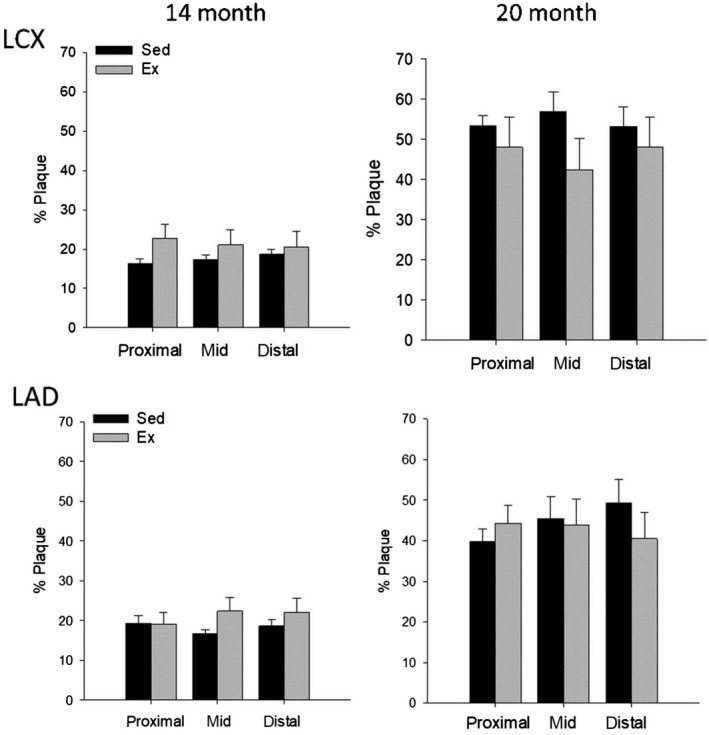
Comparison of relative plaque volume between Sed and Ex at 14 (*n* = 6 and 8, resp. for all segments) and 20 months (*n* = 8 and 8, resp. for all segments) for both the LCX (top) and LAD (bottom). There was no significant difference between Sed and Ex at 14 or 20 months in either coronary artery. Percent plaque burden = total plaque volume/vessel volume.

Prior to high‐fat, high‐cholesterol diet, 4 months of endurance exercise training resulted in a greater coronary artery size (vessel and lumen volume) in the proximal and mid sections of the LCX compared to sedentary animals (Fig. 4A and C), with no effect in the LAD (Fig. 5A and C). Prior to HFC diet, relative plaque volume was minimal (typically 15–20% vessel area) in both Sed and Ex groups (Fig. 6). After 6 months on HFC diet, there was a significant 5–6 fold increase in absolute plaque volume in all segments of the LCX and LAD in both groups. Exercise training had no effect on absolute plaque volume at 20 months in any segment of either LCX (Fig. 4F) or LAD (Fig. 5F). In both Sed and Ex groups, vessel volume was greater at 20 months compared to 14 months (Figs. 4A, B and 5A, B) such that lumen volume was maintained (Figs. 4C, D and 5C, D) despite the increase in plaque volume (Figs. 4E, F and 5E, F), reflecting a similar compensatory outward remodeling in both groups. Interestingly, in the LCX, the outward remodeling response during plaque accumulation was less in the Ex group, likely due to the prior outward expansion due to Ex alone (14 months). Overall at 20 months, there was no significant difference in vessel volume, lumen volume, absolute or relative plaque volume in either the LCX or LAD between sedentary and exercise trained animals. The only exception was a significantly greater vessel volume in the LAD (Fig. 5B), however lumen volume, absolute, and relative plaque were not different.

## Discussion

The epidemiological and human clinical trial evidence that lifestyle modifications which include increased physical activity are beneficial in treating CHD is unequivocal (Niebauer et al. 1997; Thompson et al. 2003; Sofi et al. 2008; Cui et al. 2014; Booth et al. 2017). The concept of a direct, independent effect of exercise on attenuating atherosclerosis is strengthened by putatively beneficial coronary vascular adaptations to exercise training observed in both humans and animals (Bowles et al. 2000; Laughlin et al. 2012). Atherosclerosis is an inflammatory, proliferative disease that develops over decades, and includes the involvement of numerous cell types including endothelial cells, smooth muscle cells, fibroblasts, macrophages, T‐cells, and B‐cells as well as platelets (Libby and Theroux 2005). Endurance exercise training is known to produce multiple potentially beneficial changes in the smooth muscle and endothelium of pigs with diet induced hypercholesterolemia and early‐stage atherosclerosis (Korzick et al. 2005; Laughlin et al. 2012). However, these beneficial effects in hypercholesterolemic swine are not universal. Exercise training did not attenuate the development of a pro‐atherogenic phenotype in coronary arteries of Yucatan swine fed a high‐fat, high‐cholesterol diet (Arce‐Esquivel et al. 2012). In addition, carotid endothelial VCAM‐1 expression is not decreased nor endothelium‐dependent dilation increased by exercise training in FH swine (Masseau et al. 2012). Furthermore, coronary endothelial function is depressed in FH swine and is not improved by exercise training (Simmons et al. 2014). These observations have led to the conclusion that exercise training does not produce an improved endothelial phenotype in coronary arteries of FH swine. These findings are consistent with the observations in the current study whereby exercise training did not attenuate coronary lesion development in either the LAD or LCX in FH swine. Thus, the current findings fail to support a direct, independent effect of exercise training in limiting the progression of coronary atherosclerotic lesion development in FH swine.

There are some important considerations to the current study that may limit extrapolation to CHD in humans. First, the current study used a combination of genetic familial hypercholesterolemia and high‐fat, high‐cholesterol diet to accelerate the development of clinically significant CHD from decades in humans to 6 months in swine. While this was successful in producing high relative plaque burden, with many exceeding the clinical threshold for intervention (i.e., 70% plaque burden), this also resulted in total cholesterol, LDL, and TC/HDL ratios dramatically higher than observed in the general human population at risk for CHD. This accelerated disease protocol may have resulted in a “sledge hammer” effect on atherosclerosis disease progression that overwhelmed any putative direct beneficial effect of exercise on the coronary vasculature. This concept is supported by a previous study demonstrating that exercise training was able to reduce neointimal formation following balloon angioplasty in non‐atherogenic swine (Fleenor and Bowles 2009a). In addition, exercise training reduced very early stage atheroma in hypercholesterolemic Yucatan swine (Long et al. 2010) and swine with metabolic syndrome (Edwards et al. 2010) with lower lipid burdens. Thus, the ability of exercise to mitigate advanced coronary disease may require swine models where CHD is allowed to progress to advanced stages in a more natural, long‐term progression.

In the early stages of atherosclerosis, arteries enlarge in relation to plaque area (i.e., outward or positive remodeling) to preserve lumen diameter until lesion area exceeds ~40% of vessel area an adaptive response termed Glagov's phenomenon. Approximately 60% of arteries compensate appropriately, while others fail to remodel or show excessive expansive outward remodeling which increases risk for plaque rupture. In the current study coronary arteries from both Sed and Ex exhibited compensatory outward remodeling, such that lumen diameter was maintained, despite increased plaque growth. The average plaque burden in the LAD and LCX of both groups ranged from ~40 to 55%, which is near the limit for compensatory remodeling. The outward remodeling that occurred with exercise‐training in the absence of disease and the compensatory outward remodeling that occurred in both groups during disease progression likely have similar underlying mechanisms. In both instances, outward vascular remodeling likely occurred to normalize endothelial shear stress. In exercise trained animals in the absence of disease, the increased endothelial shear stress due to increased coronary blood flow secondary to increased myocardial oxygen consumption during exercise led to outward remodeling through nitric‐oxide mediated pathways (Newcomer et al. 2011). The compensatory outward remodeling in both sedentary and exercise trained animals during disease progression also likely occurred through a similar mechanism to normalize shear stress that initially increased as plaque volume increased (Glagov et al. 1987; Korshunov et al. 2007).

Animals who were exercise trained for 4 months prior to initiation of HFC diet exhibited larger vessel and lumen dimensions in the proximal and mid sections of the LCX compared to sedentary counterparts. This evidence of exercise‐induced outward remodeling is consistent with prior studies demonstrating exercise increases coronary vasculature volume in rodents (Tepperman and Pearlman 1961) and conduit coronary artery size in dogs (Wyatt and Mitchell 1978) and monkeys (Kramsch et al. 1981). In humans, echocardiographic and MRI data indicate an increased cross‐sectional area of the left main coronary artery (Kozakova et al. 2000, 2007) while angiograms revealed a greater dilation‐induced diameter in athletes (Haskell et al. 1993). While generally increased proximal coronary artery size is observed proportional to changes in LV mass (Laughlin et al. 2012), Windecker et al. (2002) reported that a 5 month exercise training protocol in humans produced increases in proximal LAD, but not proximal LCX, cross‐sectional area (Windecker et al. 2002). Similarly, it was noted that the exercise‐induced increase in proximal and mid RCA was not present in the distal RCA. Similar non‐uniform changes in coronary artery diameter were observed in the current study. Overall, these findings are consistent with the conclusion that exercise‐induced increases on flow capacity are accomplished by a combination of structural and functional adaptation, with structural changes prominent in more proximal, conduit arteries (Laughlin et al. 2012; Bruning and Sturek 2015). Thus, it appears that the exercise training in the current study was sufficient to induce coronary vascular remodeling in the absence of disease.

There were important limitations to the current study that warrant consideration. The study design did not include determination of coronary vascular morphometry prior to initiation of the exercise training protocol, however the swine that entered the study were randomly assigned to sedentary or exercise trained groups, thus minimizing the possibility that differences in coronary artery dimensions observed after the initial 4 months of exercise training were unrelated to exercise training. The study design was also cross‐sectional, rather than longitudinal, as data from two different sedentary groups were used for the two time points. The current study also used castrated male swine, thus influences of testosterone in males and whether similar effects would be observed in females is unknown.

Prior and concurrent endurance exercise training failed to alter the progression of diet‐induced coronary atherosclerosis in a LDL receptor mutant swine model of hypercholesterolemia. Thus, the present study did not find evidence to support an independent, direct effect of exercise in limiting CHD progression in familial hypercholesterolemia.

## Conflict of Interest

None declared.
